# Optimization of Aqueous Extraction of Polyphenols from *Cuminum cyminum* Seeds Using Response Surface Methodology and Assessment of Biological Activity

**DOI:** 10.3390/biotech13010007

**Published:** 2024-03-21

**Authors:** Hana El Tannir, Diana Houhou, Espérance Debs, Mohamed Koubaa, Adla Jammoul, Bilal Azakir, Mahmoud I. Khalil, Nada El Darra, Nicolas Louka

**Affiliations:** 1 Department of Biological Sciences, Faculty of Science, Beirut Arab University, Beirut P.O. Box 11-5020, Lebanon; hana.tannir@bau.edu.lb (H.E.T.); or mahmoud_ibrahim@alexu.edu.eg (M.I.K.); 2 Department of Nutrition and Dietetics, Faculty of Health Sciences, Beirut Arab University, Beirut P.O. Box 11-5020, Lebanon; dianahuhu88@gmail.com (D.H.); n.aldarra@bau.edu.lb (N.E.D.); 3 Department of Biology, Faculty of Arts and Sciences, University of Balamand, Tripoli P.O. Box 100, Lebanon; esperance.debs@balamand.edu.lb; 4 Université de Technologie de Compiègne, ESCOM, TIMR (Integrated Transformations of Renewable Matter), Centre de Recherche Royallieu, CS 60319, CEDEX, 60203 Compiègne, France; 5 Lebanese Agricultural Research Institute, Food Department, Beirut P.O. Box 2611, Lebanon; ajammoul@lari.gov.lb; 6 Molecular and Translational Medicine Laboratory, Faculty of Medicine, Beirut Arab University, Beirut P.O. Box 11-5020, Lebanon; b.azakir@bau.edu.lb; 7 Molecular Biology Unit, Department of Zoology, Faculty of Science, Alexandria University, Alexandria 21568, Egypt; 8 Centre d’Analyses et de Recherche, Unité de Recherche Technologies et Valorisation Agro-Alimentaire, Faculté des Sciences, Saint-Joseph University, Beirut P.O. Box 17-5208, Lebanon; nicolas.louka@usj.edu.lb

**Keywords:** *Cuminum cyminum*, polyphenols, extraction optimization, response surface methodology, antimicrobial activity, anticancer activity

## Abstract

(1) Background: Cumin seeds, extracted from the plant *Cuminum cyminum*, are abundant in phenolic compounds and have been extensively researched for their chemical makeup and biological effects. The objective of this research is to enhance the water extraction of polyphenols through the water bath (WB) technique and to evaluate the antiradical, antibacterial, and anticancer effects of the extract. (2) Methods: Response Surface Methodology was used to find the best parameters to extract polyphenols. Three experimental parameters, time, temperature, and solid-liquid ratio, were tested. The disc diffusion method has been used to determine the antimicrobial activities against *Salmonella* Typhimurium, *Pseudomonas aeruginosa*, *Escherichia coli*, *Staphylococcus aureus*, and *Candida albicans*. The antiradical activity was performed using the DPPH method, while total phenolic content was performed using Folin–Ciocalteu. High-Performance Liquid Chromatography (HPLC) was conducted to analyze the phytochemical profile of WB extracts. The anticancer activity of the lyophilized extract was assessed against three cancer cell lines (colon (HT29), lung (A549), and breast (MCF7) cancer cell lines).; (3) Results: The optimal conditions for water extraction were 130 min at 72 °C. The total phenolic compounds yield (14.7 mg GAE/g DM) and antioxidant activity (0.52 mg trolox eq./mL) were obtained using a 1:40 solid–liquid ratio. The primary polyphenols identified were the flavonoids rutin (0.1 ppm) and ellagic acid (3.78 ppm). The extract had no antibacterial or antifungal activities against the microorganisms tested. The extract showed anticancer activity of about 98% against MCF7 (breast cancer cell line), about 81% against HT29 (colon cancer cell line), and 85% against A549 (lung cancer cell line) at high doses. (4) Conclusions: Extraction time and a high solid–liquid ratio had a positive impact on polyphenol recovery and in maintaining their quantity and quality. Furthermore, the optimal aqueous extract exhibited strong antiradical activity reflected by the inhibition of free radicals in addition to a significant specificity against the tested cancer cell lines.

## 1. Introduction

**Figure 1:**
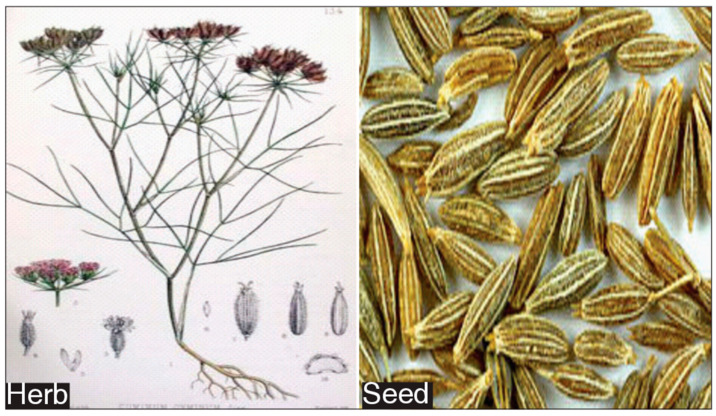
*Cuminum cyminum*. Reproduced from [2].

*Cuminum cyminum* is one of the earliest plants grown in Asia, Africa, and Europe, belonging to the *Apaiaceae* family [1]. Since antiquity, cumin seeds have been widely utilized in traditional therapy worldwide, as well as in culinary spices and herbal infusions (Figure 1) [2]. For instance, in Italy, cumin is used as a digestive aid and laxative, while it is regarded in Tunisian medicine as a blood pressure-lowering herb, a contraceptive, and lactogenic [3]. The seeds are used in most medical prescriptions as antidiarrheal medications and antiacids, as stimulants, and to relieve symptoms of bloating and discomfort in traditional Arabic medicine. Moreover, they are applied as a poultice to alleviate the pain of helminthic infection [4,5]. The health benefits of cumin seeds, such as anti-inflammatory, antibacterial, and anticancer activities, have been documented in several studies [4,6,7,8,9,10,11,12,13]. Cumin seeds are well known for being high in polyphenolic chemicals, including tannins, flavonoids, and phenolic acids, which support their range of biological functions [2,14,15,16,17]. One of the main classes of polyphenolic chemicals present in cumin seeds is flavonoids [18]. These substances, including apigenin, luteolin, and quercetin, have an antioxidant effect by eliminating harmful free radicals from the body, thereby mitigating oxidative stress on cells and tissues [19]. Additionally, flavonoids possess an anti-inflammatory activity by preventing the development of pro-inflammatory molecules that reduce inflammation and related health hazards [20]. Phenolic acids, comprising caffeic acid, ferulic acid, and *p*-coumaric acid, are highly concentrated in cumin seeds and are known to have antioxidant and antitumor activities, to prevent heart disease and control blood sugar [6,15,19,21].

The concentration of polyphenols in cumin seeds might vary based on the plant variety, growth environment, and processing techniques. Research has demonstrated that factors including seed maturity, extraction methods, and storage conditions can affect the polyphenol concentration of cumin seeds [22]. Various solvents and extraction methods were used to evaluate polyphenols’ content and to assess the antioxidant activity of extracts from plant sources [23,24,25,26,27,28,29]. These extraction techniques include conventional methods such as the use of a water bath [30], the use of diverse solvents such as hexane, methanol, or ethanol [31,32], or the use of microwave-assisted extraction (MAE) [25], supercritical fluid extraction or Soxhlet extraction [24], infrared assisted extraction [33,34,35] and ultrasonic-assisted extraction (UAE) [36]. Furthermore, many variables, including extraction technique, duration, and temperature, either separately or in combination [22,24,25,28,37,38,39], could have a considerable impact on the yield and efficiency of the process. Therefore, an optimization for time and temperature for the extraction process is required to maximize the yield especially when a number of factors could affect the output. A set of quantitative tools called response surface methodology (RSM) is useful for streamlining and optimizing procedures, examining the relationships between the response and independent variables, and predicting the response [39]. Hence, the goal of this research was to improve the aqueous extraction of polyphenols in the function of time and temperature from cumin seeds using the conventional water bath method in order to simulate the use of *C. cyminum* as an infusion for human consumption using RSM and to assess the efficiency of the extract in neutralizing free radicals, inactivating bacteria, and inhibiting the growth of cancer cells.

## 2. Materials and Methods

### 2.1. Plant Material

*C. cyminum* was bought from a local Lebanese market that imports seeds from Syria. Before the extraction process, *C. cyminum* seeds were crushed with a blade grinder and kept in a dark and cold place for later use.

### 2.2. Chemicals, Reagents, Media, and Microorganisms

#### 2.2.1. Chemicals and Reagents

All the chemicals and solvents needed for the experiments, including trolox (6-hydroxy-2,5,7,8-tetramethylchromane-2-carboxylic acid), Folin–Ciocalteu reagent, gallic acid (3,4,5-trihydroxybenzoic acid), dimethylsulfoxide (DMSO; HPLC grade), sodium carbonate (Na_2_CO_3_), and DPPH (2,2-diphenyl-picrylhydrazyl), in addition to standards for HPLC, were acquired from Sigma-Aldrich (Steinheim, Germany).

#### 2.2.2. Media and Microorganisms

Mueller–Hinton Agar (MHA) and Mueller–Hinton Broth (MHB) were bought from HIMEDIA (Mumbai, India) and prepared according to the manufacturer’s instructions. As for microorganisms utilized in this research, one Gram-positive strain, three Gram-negative strains, and one fungus were acquired from the microbiology laboratory at the Lebanese Agricultural Research Institute, Fanar, Lebanon. These microorganisms are listed in Table 1 and were employed to ascertain the antimicrobial effect of *C. cyminum* extracts. Strains were preserved at -80 °C in glycerol until they were utilized.

### 2.3. Dry Matter in C. cyminum

The amount of dry matter (DM) in the cumin seeds was measured by placing the seeds in an oven ventilated at a temperature of 105 °C for 24 h. The amount of DM was then estimated, and the results were stated as a percentage of the total weight of the seeds. The dry matter content of cumin seeds was 88.7 ± 0.2% *w*/*w*.

### 2.4. The Extraction Procedure

Various solid-to-liquid ratios ranging from 1/10 to 1/50 (*w*/*v*) were utilized for the extraction procedure. Response surface methodology (RSM) was then used to optimize the extraction process after establishing the solid-to-liquid ratio corresponding to the best TPC yield. A volume of 200 mL water served as the solvent, and five grams of pulverized *C. cyminum* seeds were added at a solid-to-liquid ratio of 1/40. After filtering the extracts, the filtrates underwent a 10 min centrifugation at 5000 rpm and were then stored at −18 °C.

A water bath shaker (DKZ-1 series) was used to perform the Water Bath extraction (WB). Using distilled water as the solvent, ground *C. cyminum* seeds were added to an Erlenmeyer flask and stirred for the specified amount of time at the proper temperature.

### 2.5. Experimental Design

Several factors influence the number of total polyphenols and their quality. To assess the impact of each parameter and the interactions between the factors, an optimization of the extraction process using RSM was conducted. The core concept of RSM involves systematically conducting a series of designed experiments wherein the levels of independent variables are intentionally varied within predetermined ranges. For each combination of input variable levels, the response variable is measured, resulting in a dataset that captures the behavior of the system across a diverse set of conditions. These experimental outcomes are then employed to construct empirical models that portray the relationship between the input variables and the response variable.

At the heart of RSM lies the creation of a response surface, which can take the form of a graphical representation or a mathematical equation illustrating the correlation between the independent variables and the response variable. This response surface serves as a tool for understanding optimal conditions that produce the desired response and aids in pinpointing critical factors that influence the system’s performance.

The mathematical models derived through RSM typically take the form of polynomial equations, offering a means to describe the system’s behavior within the designated experimental domain. These models enable the prediction of the response variable for untested combinations of input variables, which helps to identify ideal operating conditions and make informed decisions to achieve specific outcomes [40,41,42,43].

In this study, RSM was conducted considering the extraction time as “t” and the temperature as “T”. To evaluate the impact of the extraction time and temperature on TPC and DPPH concentration as response variables, a central composite design of experiments with twelve runs and four repetitions at the central points was created. The temperature ranged from 31.7 °C to 88.2 °C, while the extraction time ranged from 11.55 min to 138.45 min. The highest and lowest values were considered to have levels of −α and +α, respectively. The design of all experiments and the findings were analyzed using STATGRAPHICS Centurion XVII-X64.

### 2.6. Measurement of the Total Phenolic Content

The Folin–Ciocalteu method was employed to calculate TPC, as previously indicated [44]. A volume of 100 μL of *C. cyminum* extract was combined with 500 μL of Folin–Ciocalteu reagent (diluted 1/10 *v*/*v*) and 400 μL of Na_2_CO_3_ 7.5% (*w*/*v*). The mixture was incubated for 10 min at 60 °C and then cooled down for 10 min at 4 °C. Referring to a UV-vis spectrophotometer (GENESYS 10 UV, Thermo Electron Corporation, Waltham, MA, USA), the measurement for the absorbance was performed at 750 nm and the standard, gallic acid, was utilized to determine the calibration curve. TPC was measured in milligrams of gallic acid equivalents per gram of dry matter (mg GAE/g DM).

### 2.7. Antiradical Activity

The antiradical effect of the extracts was assessed by their capacity to reduce the free radical DPPH (2,2-diphenyl-picrylhydrazyl) [45]. Next, 1.45 mL of DPPH solution (0.06 mM) was added to 50 μL of *C. cyminum* extracts or trolox (as positive control). The mixture was incubated at room temperature for 30 min in the dark and then a measurement for the absorbance was performed at 515 nm. The antiradical activity of the extracts was calculated by comparing their absorbance to the absorbance of the blank (pure methanol) using the following equation:

$$Inhibition\;percentage=\frac{Absorbance\;of\;negative\;control-Absorbance\;of\;sample}{Absorbance\;of\;negative\;control}\times 100$$

The antiradical activity was measured in micrograms of trolox equivalent per milliliter (μg TE/mL).

### 2.8. High-Performance Liquid Chromatography Analysis

The extracts from *C. cyminum* using the WB method under optimal conditions were interpreted using high-performance liquid chromatography (HPLC). An Agilent 1100 series HPLC system with a Zorbax column oven (Barcelona, Spain), an autosampler, and a diode array detector were utilized for this investigation (Teknokroma Professional Friendly Lichrospher 100 RP18 5 mM, 25 × 0.46, Serial Number NF-21378, Barcelona, Spain). Phenolic chemicals were separated using a C18 column (25 × 0.46 mm). The following chemicals were used as standards to identify and quantify the phenolic compounds: gallic acid, hydroxybenzoic acid, protocatechuic acid, catechin, chlorogenic acid, caffeic acid, *p*-coumaric acid, rutin, ellagic acid, trans-cinnamic acid, and quercetin (Sigma-Aldrich, Steinheim, Germany). The mobile phase was composed of acidified purified water and methanol with a pH of 2.3. Under isocratic conditions, the elution procedure was carried out using 85% acidified purified water and 15% methanol from 0 to 5 min. Then, from 5 to 30 min, a gradient profile was used, changing from 85% acidified purified water and 15% methanol to 0% acidified purified water and 100% methanol. Then, isocratic conditions with 0% acidified purified water and 100% methanol were followed for 30 to 35 min. The flow rate was set at 1 mL/min, and the injection volume was 10 µL. Phenolic compounds were identified based on a comparison of the retention times of the observed peaks with those of the original reference compounds. The concentration of phenolics was calculated by creating standard curves for each individual compound using various amounts of the relevant standards.

### 2.9. Antibacterial and Antifungal Activity Assay

#### 2.9.1. Preparation of the Inoculum

For the antibacterial and antifungal activities, isolated bacterial colonies with known ATCC were suspended in sterile saline at a density corresponding to that of 0.5 McFarland standards (prepared by mixing 0.05 mL of 1.175% barium chloride dehydrate with 9.95 mL of 1% sulfuric acid).

#### 2.9.2. Antibacterial and Antifungal Activities Using Disc Diffusion Method

Using a sterile cotton swab, a microbial suspension (equivalent to a 0.5 McFarland standard) was spread over Mueller–Hinton agar plates. Then, blank sterile filter discs (6 mm in diameter) were impregnated with 20 μL of cumin seed extract and aseptically placed on the surface of the inoculated agar. The positive control was considered the disc impregnated with 20 μL of standard antibiotic gentamicin (MAST Co., Liverpool, UK), and the disc impregnated with distilled water was considered as negative control. The Petri dishes were then incubated at 37 °C for 24 h. The diameters of the inhibition zones around the discs were measured after the incubation period. This experiment was performed in triplicates.

#### 2.9.3. Determination of the Minimum Inhibitory Concentration and the Minimum Bactericidal Concentration

The minimum inhibitory concentration (MIC) of *C. cyminum* extract was determined using the broth macrodilution method [46]. An adjustment of the bacterial suspension to 0.5 McFarland was conducted. Then, a volume of 1 mL of Mueller–Hinton broth (MHB) was added in 5 tubes. Afterwards, 1 mL of the extract with a starting concentration of 25 mg/mL was added to the first tube and serially diluted (12.5, 6.25, 3.1, 1.55 mg/mL). Then, 1 mL of the bacterial strain was added to all tubes. The broth and bacteria served as a positive control tube, while the negative controls were broth alone and broth with extract. Following 24 h of incubation at 37 °C, the MIC was detected as the lowest concentration that inhibited the growth of the bacteria.

To determine the minimum bactericidal concentration, 100 µL of each tube was added to MHA plates and distributed on agar with a sterile rod. The negative control was 100 µL of extract with broth, and the positive control consisted of 100 µL of broth and bacteria. An incubation was followed for 24 h at 37 °C for the plates. The plate that displays zero colonies is considered the minimal bactericidal concentration (MBC).

### 2.10. Determination of In Vitro Anti-Tumor Activity of C. cyminum Seeds Extracts

#### 2.10.1. Cell Culture and Treatment

Colon cancer cell line (HT-29), lung cancer cell line (A549), and breast cancer cell line (MCF-7) were utilized to evaluate the anti-tumor activity of the extracts. HT-29, A549, and MCF-7 cells were cultured in RPMI-1640 (Sigma-Aldrich), suited for the cultivation of cells derived from the blood containing essential nutrients, vitamins, amino acids, and salts that support cell growth. Dubelcco’s modified Eagle’s Media (DMEM) Ham’s F-12 (Sigma-Aldrich), offering a balanced and versatile environment suitable for cell lines, and DMEM-high glucose, providing a rich nutrient base including amino acids and vitamins to support cell proliferation (Sigma-Aldrich), were supplemented respectively with 10% heat-inactivated fetal bovine serum (FBS) (Sigma-Aldrich) and 1% penicillin/streptomycin (Sigma-Aldrich), and incubated at 37 °C with 5% CO_2_.

#### 2.10.2. MTT Assay

The antiproliferative effects of *C. cyminum* seed extracts were measured in vitro using 3-(4,5-dimethylthiazol-2-yl)-2,5 diphenyltetrazolium bromide (MTT) assay according to the manufacturer’s instructions (Sigma-Aldrich). Briefly, cells were seeded (6 × 10^3^ cells/well) in 100 µL complete medium of cell suspension, DMEM, and FBS in 96-well plates and incubated for 72 h at 37 °C with 5% CO_2_. After incubation, MTT was added, and the plates were incubated again for 4 h. Then, a solubilizing agent, isopropanol, was added to each well and incubated in a shaker for 15 min. The absorbance was read at 595 nm using the ELISA Reader (Thermo Scientific, Waltham, MA, USA). The effect of cumin extracts on cancer cell growth was determined by MTT assay. Cells were seeded in 96-well plates at a concentration of 6 × 10^3^ cells/well. After 24 h, the seeded cells were treated with various concentrations of seed extracts for 72 h. After treatment, cells were incubated with 5 mg/mL of MTT for 4 h, and the reaction was stopped with 100 μL isopropanol added per well. A measurement for the absorbance at 595 nm was performed using an automatic microplate reader. Each condition was performed in triplicate. The treated cells were compared to the control cells, and the percentage (%) of viability was calculated according to the following equation:

$$\%\;viability=\frac{{Absorbance}_{\;treated\;cell}\;}{{Absorbance}_{\;Control}}\times 100$$

### 2.11. Statistical Analysis

Each experiment and measurement was repeated three times, and the results were expressed as the average value plus or minus the standard deviation. Analysis of variance (ANOVA) was employed to compare data between the control and the experimental groups through the assessment of p-values. Statistical significance was attributed to p-values less than 0.05, indicating a confidence level of 95%. The optimization of the extraction process was conducted using STATGRAPHICS^®^ Centurion XVII-X64 software as part of the statistical analysis.

## 3. Results and Discussion

### 3.1. Selection of Solid to Liquid Ratio

The solid-to-liquid ratios of 1/10, 1/20, 1/30, 1/40, and 1/50 were examined (Table 2). Values are shown as means of standard deviations from triplicate measurements. The total phenolic content (TPC) of the samples was not significantly affected by the different extraction ratios, and the optimal extraction ratio was chosen to continue the subsequent experiments according to the optimal polyphenolic content obtained, which is a 1/40 ratio with a TPC of 13.44 mg GAE/g DM. Similar to these results, Zhang et al. [47] reported that in microwave-assisted extraction, the yields of six phenolic compounds increased with a decrease in solid-to-liquid ratios. However, the authors did not observe any significant difference in the yields obtained with ratios ranging from 1/30 to 1/50. Patil et al. [48] cited an optimum polyphenol extraction from curry leaves while using a solid-to-solvent ratio of 1/40 (g/mL). In contrast, Pinelo et al. [49] observed that utilizing lower solvent-to-solid ratios resulted in higher total polyphenol values from grape pomace.

### 3.2. Influence of Time and Temperature on TPC Yield and DPPH Inhibition Percentage

Response surface methodology was adopted to optimize the extraction of polyphenols in order to choose the best parameters for the optimum yield of extraction and the optimum antiradical activity for the water bath technique. The optimum solid-to-liquid ratio was set to 1/40 (*w*/*v*), and a model was created by adjusting the time and temperature. Table 3 lists the values of the total phenolic content (mg GAE/g DM) and trolox equivalent (mg/mL) for the water bath extracts.

**Figure 2:**
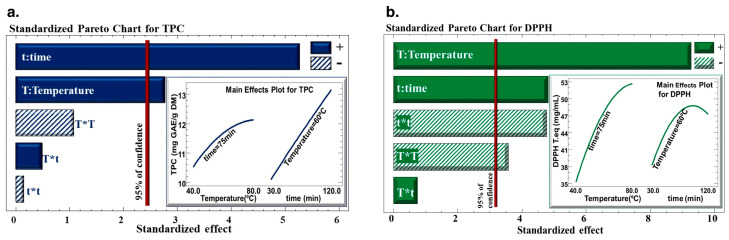
Pareto Chart for (**a**) total phenolic content (TPC) and (**b**) DPPH inhibition percentage. The sign (+) indicates a positive effect, and the sign (−) indicates a negative effect.

The Pareto charts (Figure 2) highlight how the time and the temperature of the extraction process affect the total phenolic content (TPC) of the extract and DPPH in *C. cyminum* seed extract. The results revealed that the time and temperature of extraction had a positive linear effect on the water bath extraction technique’s ability to extract polyphenols from *C. cyminum* seeds, and their DPPH inhibition. However, quadratic effects of temperature and time negatively affected the antiradical activity of the extract.

The response surface methodology was used to optimize the recovery of polyphenols after choosing the best solid-to-liquid ratio (1/40; *w*/*v*). Time and temperature had a positive linear impact on TPC because they increased the yield of TPC to its highest level. According to numerous studies [33,35], raising the temperature enhances the output of extraction by facilitating mass transfer by increasing the solubility of the solute and the diffusion coefficients. These results align with research by Pinelo et al., highlighting that the efficiency of extraction was discovered to be significantly influenced by temperature and the ratio of solvent to solid, whereas values of 50 °C (between 25 and 50 °C) and using a 1/1 ratio (between 1/1 and 5/1) increased the antiradical activity of total phenolic compounds of grape byproducts [49].

Moreover, following a high extraction temperature, a rupture may occur in the cell walls, which will lead to the release of polyphenols. The fact that some phenolic compounds may be lost or damaged when high extraction temperatures are combined with prolonged periods of time is what accounts for the adverse quadratic effect of time on TPC [37].

As for the antiradical activity, temperature and time had negative quadratic effects. Despite the fact that a greater extraction temperature increases TPC yield linearly, the heating process degrades and/or oxidizes phenolic compounds, which has a negative impact on their quality.

**Figure 3:**
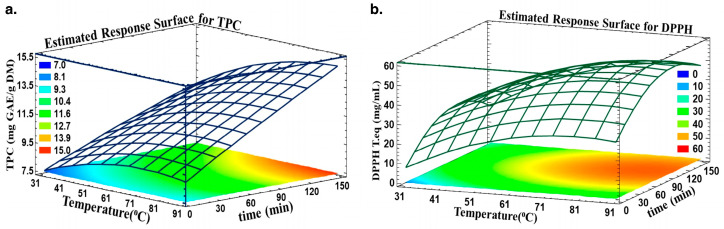
Predicted Response Surfaces for (**a**) TPC and (**b**) DPPH as a function of time and temperature.

Estimated Response Surface was adopted to highlight the optimum time and temperature for optimal TPC and antiradical activity. Figure 3 shows the TPC (a) and DPPH (b) in relation to time and temperature. The optimal area is colored orange, where any time and temperature in this designed region will yield approximately 14.7 mg GAE/g DM and an antiradical concentration of 0.52 mg trolox eq./mL.

Table 4 presents the second-degree model equations, as generated by RSM, allowing us to predict the response values of TPC and DPPH.

### 3.3. Optimization of Extraction

**Figure 4:**
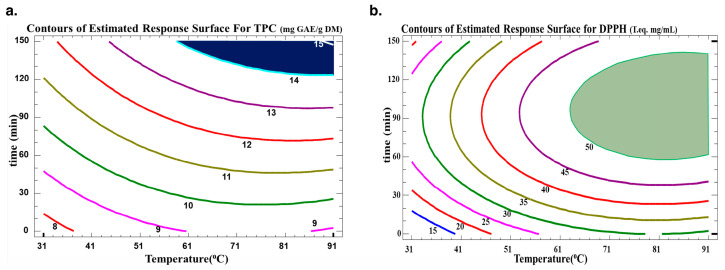
Contours of the estimated response surface for (**a**) TPC and (**b**) DPPH in the function of time and temperature for *C. cyminum* water bath extracts.

**Figure 5:**
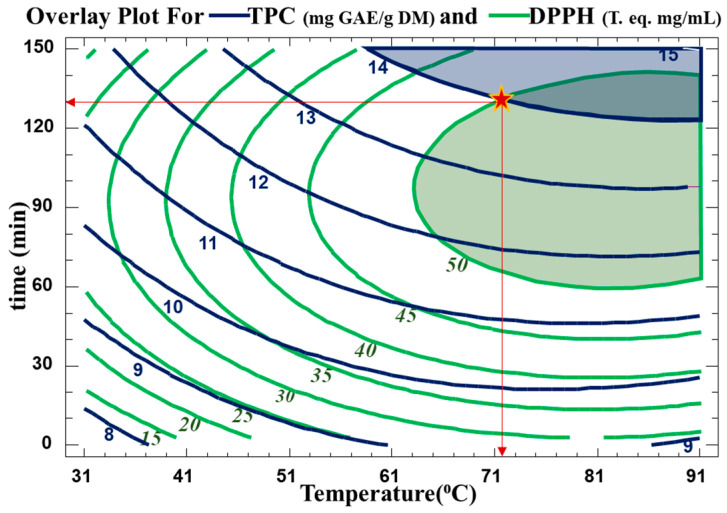
Overlay plot showing optimum condition regions (time, temperature) for maximum TPC and trolox eq. The star (*) represents the optimum values used in this study.

The optimum extraction conditions for the WB technique are presented in Table 5. Multiple response optimization was used to maximize both the number of polyphenols and their antiradical activity at the same time. The optimal conditions found to maximize TPC yield in the extract while preserving their antioxidative activity were 130 min at 72 °C. The contour plots of the estimated response surface for TPC as a function of time and temperature and for trolox equivalence in relation to time and temperature for the *C. cyminum* WB extracts are shown in Figure 4. As indicated in Table 5, high R^2^ values (R-squared for TPC of 90 and R-squared for DPPH of 94) for the model indicated an acceptable degree of adequacy between it and the experimental findings. As indicated, the optimum TPC is between two contour lines of TPC values of 14 and 15 mg GAE/g DM. Therefore, for a value of 14.57 mg GAE/g DM, the optimal conditions were around 88.2 °C and 138 min. This optimum was found within the domain of variation of the parameters chosen for a temperature range from 31.7 °C to 88.2 °C and a time range from 11.5 min to 138.7 min. As for the antiradical concentration, an optimum of 0.54 mg trolox eq./mL can be obtained with water bath extraction at 84.3 °C for 99.4 min. The regions obtained from the optimization for TPC and DPPH scavenging activity are plotted in Figure 5.

In order to confirm the model’s potential for prediction, the extraction was carried out under the expected ideal conditions. TPC and DPPH (Table 5) were examined, and experimental results supported the predicted values.

### 3.4. Antibacterial Activity

For the purpose of testing the antimicrobial activity of *C. cyminum* seed extracts, a disc diffusion method was used, followed by minimum inhibitory concentration and minimal bactericidal concentration. The results did not reveal an inhibitory activity for the extract against *C. albicans*, *S.* Typhimurium, *E. coli, S. aureus*, and *P. aeruginosa*. For MIC and MBC, bacterial growth was observed in all tubes and plates, respectively, suggesting that the aqueous extract had no antibacterial or antifungal effect of the aqueous extract. The absence of antimicrobial activity could be due to several factors, such as the extraction method, noting that in this study, water was considered the extracting solvent. Moreover, the origin of the *C. cyminum* used and its storage conditions are also the parameters that affect the antimicrobial effect of the extract. A study by Goswami et al. [12] conducted on ethanolic extracts of *C. cyminum* and water extracts showed that the ethanolic extract had antimicrobial activity against *S. aureus* and *Klebsiella pneumoniae*, and the aqueous extract of *C. cyminum* also showed good antibacterial activity against *S. aureus*, while moderate antibacterial activity was observed against *Klebsiella pneumoniae*, and was absent against *E. coli*. Additionally, the effectiveness of the concentration of *C. cyminum* water extract may vary depending on the specific bacteria or fungi targeted.

### 3.5. Antitumor Activity of the Optimal Conditions for WB Extracts

**Figure 6:**
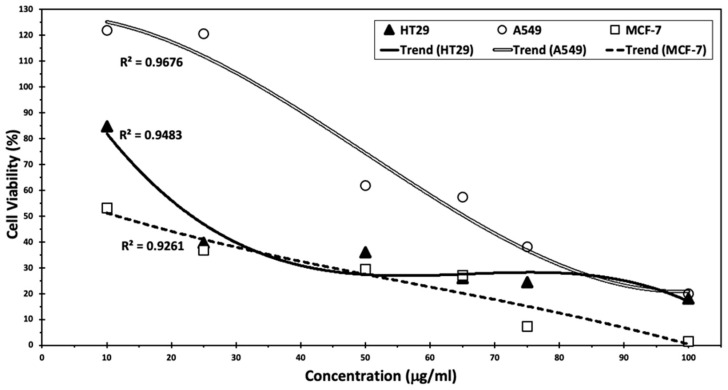
Anti-tumor activity of cumin seed extracts on colon cancer cell line HT29, lung cancer cell line A549, and breast cancer cell line MCF-7.

Cumin seed extract was able to inactivate cells dependent on the dosage. The strongest effect was observed with MCF-7 cells (2%) with an IC_50_ of 11.7 µg/mL, followed by A549 (15%) with an IC_50_ of 60.5 µg/mL and HT-29 (19%) with an IC_50_ of 23.5 µg/mL with 100 µL of extracts (Figure 6).

According to a previous study [9], cumin proved to be effective in lowering cancer cell viability, with the most pronounced effect observed on breast cancer cells, showing more than 98% inhibition. In this study, cumin silver nanoparticles in MCF-7 cells exhibited a 95% inhibition at the highest levels tested. Moreover, Goodarzi et al. highlighted the effect of *C. cyminum* mediated by the flavonoids, particularly luteolin-7-O-glucosid, making it a candidate for chemopreventive and chemotherapeutic medication [8], whereas this flavonoid demonstrated several potent anticancer activities, especially against MCF-7 cell line with an IC_50_ of 3.98 µg/mL. Furthermore, these results are aligned with those obtained by Arun et al. [7], who demonstrated the effect of *C. cyminum* extracts in enhancing apoptosis in HT29 cancer cells. Future investigations could delve into the exploration of flow cytometry and Real-Time PCR for apoptosis assay. Additionally, the assessment of gene expression related to specific signaling pathways could be conducted. Furthermore, a complete phytochemical and cytotoxic investigation of the *C. cyminum* extract might be further tested.

### 3.6. High-Performance Liquid Chromatography (HPLC) Analysis

Rutin (0.1 ppm) and ellagic acid (3.78 ppm) were the two main polyphenols found in *C. cyminum* after water bath extraction by HPLC analysis. These findings are not in compliance with those reported by Bouhenni et al. [50], where results of HPLC analysis of cumin seeds of Syrian origin extracted using 70% methanol as a solvent showed that eight phytochemical compounds were detected in cumin extract. This is mainly due to the different types of solvents used for the extraction.

Moreover, several studies emphasized the role of rutin as a potent antiradical through the neutralization of free radicals and shielding cells from oxidative stress [51,52] and documented the anti-tumor effect of ellagic acid against several cancer cell lines, highlighting its anti-inflammatory properties, antiradical activity, and modification of signaling pathways involved in cell growth and survival [53,54,55,56].

## 4. Conclusions

Cumin seeds, obtained from the *C. cyminum* plant, are known for their high content of phenolic compounds, extensively researched for their chemical composition and biological effects. The objective of this study was to optimize the extraction of polyphenols using the water bath (WB) method and to assess the resulting extract’s antioxidant, antibacterial, and anticancer properties. Response Surface Methodology guided the identification of optimal polyphenol extraction conditions, considering three experimental parameters: time, temperature, and solid-liquid ratio. The optimal extraction conditions were identified as 130 min at 72 °C, using water as the solvent, with a solid–liquid ratio of 1:40. High-Performance Liquid Chromatography (HPLC) was employed for the phytochemical profiling of WB extracts revealing the presence of the flavonoids rutin (0.1 ppm) and ellagic acid (3.78 ppm). In addition, following the Folin–Ciocalteu method and the DPPH method, results indicated a total phenolic content of 14.7 mg GAE/g DM and an antioxidant activity of 0.52 mg trolox eq./mL, respectively. The extract exhibited no antibacterial or antifungal activities against the tested microorganisms but demonstrated potent anticancer effects, with approximately 98% efficacy against MCF7, 81% against HT29, and 85% against A549 at higher doses. The study emphasizes the effectiveness of prolonged extraction times and higher solid-liquid ratios in preserving the quantity and quality of recovered polyphenols. Furthermore, the optimized aqueous extract displayed robust antiradical activity, as indicated by radical inhibition, along with notable specificity against the examined cancer cell lines. These findings shed light on *C. cyminum* extracts as antiradical and anti-tumor and as alternatives to synthetic antiradicals and anti-tumor drugs. However, further clinical and toxicological research is required to fully assess the true therapeutic potentials of polyphenols obtained from *C. cyminum*.

## Figures and Tables

**Table 1:** Microorganisms used in this study. ATCC: American Type Culture Collection.

Microorganism		Reference
Gram-positive bacteria	*Staphylococcus aureus*	ATCC 25923
Gram-negative bacteria	*Salmonella* Typhimurium	ATCC 14028
	*Escherichia coli*	ATCC 25922
	*Pseudomonas aeruginosa*	ATCC 27853
Fungus	*Candida albicans*	ATCC 10231

**Table 2:** Average total phenolic content (TPC) for different solid/liquid ratios.

Solid/Liquid Ratio (g/mL)	Average TPC (mgGAE/g DM)
1/10	12.15 ± 0.5
1/20	11.56 ± 1.6
1/30	11.29 ± 1.8
1/40	13.443 ± 3.4
1/50	12.083 ± 4.1

**Table 3:** Central composite design for the independent variables and their corresponding responses related to total phenolic content (TPC) and trolox equivalent using the water bath (WB) extraction.

Runs	Variables		Responses	
	Temperature (°C)	Time (min)	TPC (mg GAE/g DM)	Trolox Equivalent (mg/mL)
1	31.7	75	10.43	0.29
2	40	30	14.24	0.28
3	40	120	10.97	0.3
4	60	11.55	9.31	0.27
5	60	75	11.11	0.46
6	60	75	11.07	0.5
7	60	75	12.08	0.34
8	60	75	12.42	0.35
9	60	138.45	14.7	0.48
10	80	30	10.45	0.45
11	80	120	13.09	0.5
12	88.2	75	12.38	0.51

**Table 4:** Second-order regression equations for water bath extraction. TPC: Total Phenolic Content; DPPH: (2,2-diphenyl-1-picrylhydrazyl).

**TPC**	4.426 + 0.128*T + 0.024*t − 0.0009*T^2^ + 0.0002*T*t − 0.00002*t^2^
**DPPH**	−0.297 + 1.497*T + 0.420*t − 0.009*T^2^ + 0.0008*T*t − 0.002*t^2^

**Table 5:** Optimum extraction conditions for the water bath technique.

**Parameters**	**Optimum Conditions**	
	**TPC**	**DPPH**
Time (min)	138	99.4
Temperature (°C)	88.2	84.3
TPC (mg GAE/g DM)	14.57	-
DPPH (mg trolox eq./mL)	-	0.54
R-squared (R^2^)	90	94
**Parameters**	**Multiple Optimization**	
Time (min)	133	
Temperature (°C)	87	
TPC predicted (mg GAE/g DM)	14.3	
TPC observed (mg GAE/g DM)	14.7	
DPPH predicted (mg trolox eq/mL)	0.51	
DPPH observed (mg trolox eq/mL)	0.52	

## Data Availability

Data are contained within the article.
